# Influence of Anions on the Antibacterial Activity and Physicochemical Properties of Different-Sized Silver Nanoparticles

**DOI:** 10.3390/molecules29174099

**Published:** 2024-08-29

**Authors:** Bojie Yuan, Shuyue Shangguan, Deqiang Zhao

**Affiliations:** 1 Key Laboratory of Hydraulic and Waterway Engineering, Ministry of Education, Chongqing Jiaotong University, Chongqing 400074, China; shangguanshuyue@mails.cqjtu.edu.cn; 2 Department of Materials and Environmental Chemistry, Stockholm University, SE-106 91 Stockholm, Sweden

**Keywords:** silver nanoparticles, anions, particle size, antibacterial activity, *Escherichia coli*

## Abstract

Silver nanoparticles (AgNPs) with different sizes have been extensively adopted in various commercial products, causing ecological concerns because of the inevitable release of AgNPs into the environment. Hence, understanding the interaction of different-sized AgNPs with environmental substances is important for assessing the environmental risk and fate of AgNPs. In this work, we investigated the impact of anions (NO_3_^−^, SO_4_^2−^, HCO_3_^−^/CO_3_^2−^, Cl^−^) in aquatic environments on the physicochemical properties and antibacterial activity of different-sized AgNPs (20, 40 and 57 nm). The results showed that the anions whose corresponding silver-based products had lower solubility were more likely to decrease the zeta potential (more negative) of particles, inhibit the dissolution of AgNPs and reduce their antibacterial activity. This should be attributed to the easier generation of coating layers on the surface of AgNPs during the incubation process with such anions. Additionally, the generation of coating layers was also found to be particle-size dependent. The anions were more prone to adsorbing onto larger-sized AgNPs, promoting the formation of coating layers, subsequently resulting in more pronounced variations in the physicochemical properties and antibacterial activity of the larger-sized AgNPs. Therefore, larger-sized AgNPs were more prone to experiencing specific effects from the anions.

## 1. Introduction

Nanomaterials possess excellent physicochemical properties (such as macroscopic quantum tunneling effects, quantum-size effects and surface effects), which have made them attract widespread attention for a long time [1]. Recently, AgNPs have been extensively adopted in various fields, such as cosmetics, food technology and textiles/fabrics [2]. Furthermore, AgNPs have also demonstrated significant potential for application in agriculture [3], in the aquaculture industry [4,5] and as marine antifouling agents [6]. As a result, AgNPs have become one of the most commonly utilized nanomaterials [7]. However, during the production, useful life, or final disposal of AgNPs products, AgNPs are inevitably released into the environment, especially aquatic environments [8,9,10,11]. Numerous studies have shown that AgNPs exhibited adverse effects on various natural organisms (including Lolium multiflorum [12], *Microcystis aeruginosa* [13,14], *Paramecium caudatum* [15] and zebrafish [16,17]), and even human cells [18,19]. As a result, increasing concern is being raised about the possible risks to human and ecological health induced by the release of AgNPs.

Once released into the environment, it is inevitable that AgNPs will interact with various environmental factors, such as organic substances, dissolved oxygen and electrolytes, among others. Therefore, understanding the transformation of AgNPs in the natural environment is crucial for assessing their potential risks and fate in the environment. Much effort has been devoted to studying the impact of these environmental factors on the physicochemical properties, behavior, and toxicity of AgNPs [20,21,22,23,24,25,26,27,28,29,30]. Anions, which are ubiquitously present in aquatic environment, were found to be among the most important environmental factors affecting the behavior and fate of AgNPs. After exposure to S^2−^, Ag_2_S layer was proven to generate on AgNPs surfaces, which significantly inhibited the dissolution of AgNPs and markedly reduced their toxicity [24,25,26]. In Cl^−^-containing water, the transformation of AgNPs was found to be strongly associated with the ratio of Cl/Ag [27]. Under low Cl/Ag ratios, the major transformation product was AgCl layer coated on AgNPs surface, which greatly inhibited the dissolution and toxicity of AgNPs [27,28]. However, the dissolution of AgNPs was promoted under high Cl/Ag ratios, as a result of the formation of soluble AgCl_x_^(x−1)^ species, which led to an enhancement of toxicity [27]. During incubation with HCO_3_^−^, Ag_2_CO_3_ layer was observed to form on AgNPs surfaces, resulting in an increase in the surface charge (which became more negative) and a reduction in the toxicity of AgNPs [29]. Additionally, the generation of Ag_2_CO_3_ layer was found to be influenced by ambient temperature, and an increase in temperature facilitated the formation of the Ag_2_CO_3_ layer [29]. It is evident that anions in aquatic environments exhibit a significant impact on the transformation of AgNPs. However, different anions possess distinct properties, such as differences in the solubility product (*K_sp_*) of the silver compounds to which they correspond. These differences might lead to diverse effects of anions on the physicochemical properties and toxicity of AgNPs. Therefore, elucidating the correlation between AgNPs transformation and anion characteristics can provide additional insights into the behavior and potential risks of AgNPs in aquatic environments.

In addition, the inherent characteristics of AgNPs, such as particle size, may also be considered as an important factor influencing their transformation in aquatic environments. AgNPs with a decreased size show an increased specific surface area, leading to heightened reactivity and surface energy. Furthermore, it was demonstrated that the standard redox potential of AgNPs decreased as the particle size decreased [31]. Hence, in aquatic environments, smaller-sized AgNPs were more prone to oxidation and dissolution [32,33]. Additionally, it was also found that different-sized AgNPs possessed distinct optical properties. Smaller-sized AgNPs absorbed light with shorter wavelengths and higher energy [30,33], implying higher electron density on their surface [34]. Hence, there are differences between the physicochemical properties of different-sized AgNPs, potentially leading to variations in the transformation of AgNPs under the influence of anions. Nonetheless, as far as we know, the impact of particle size on the interactions of AgNPs with anions remains largely unclear.

In this study, four typical anions (NO_3_^−^, SO_4_^2−^, HCO_3_^−^/CO_3_^2−^, Cl^−^) in aquatic environments were selected to study their impact on different-sized AgNPs (20, 40, 57 nm). During the long-term incubation of AgNPs with these anions, the changes in the physicochemical properties (zeta potential, dissolution) and antibacterial activities of different-sized AgNPs were investigated. Next, the mechanism of the interaction between the anions and different-sized AgNPs was further explored. The results indicated that the interaction of AgNPs with anions was affected by both the anion characteristics and the particle size. These findings may provide valuable insights into understanding the behavior and potential hazards of AgNPs in aquatic environments.

## 2. Results and Discussion

### 2.1. Characterization of AgNPs

**Figure 1:**
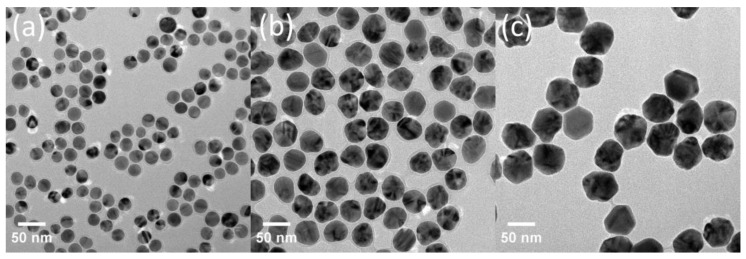
TEM images of synthesized AgNPs: ((**a**) AgNP−20, (**b**) AgNP−40, (**c**) AgNP−57).

According to the TEM images in Figure 1, all the synthesized AgNPs were quasi-spherical and exhibited monodispersity. The TEM size distributions of the synthesized AgNPs, as statistically determined by measuring the sizes of over 200 particles for each AgNPs type, were found to be 20 ± 6 nm (AgNP-20), 40 ± 6 nm (AgNP-40) and 57 ± 5 nm (AgNP-57), respectively. Furthermore, the zeta potential values of AgNP-20, AgNP-40 and AgNP-57 were measured as −7.6 mV, −9.6 mV and −10.2 mV, respectively, which were consistent with the zeta potential values reported in many previous studies for PVP-coated AgNPs [24,27,35,36].

### 2.2. AgNPs in Ultrapure Water

**Figure 2:**
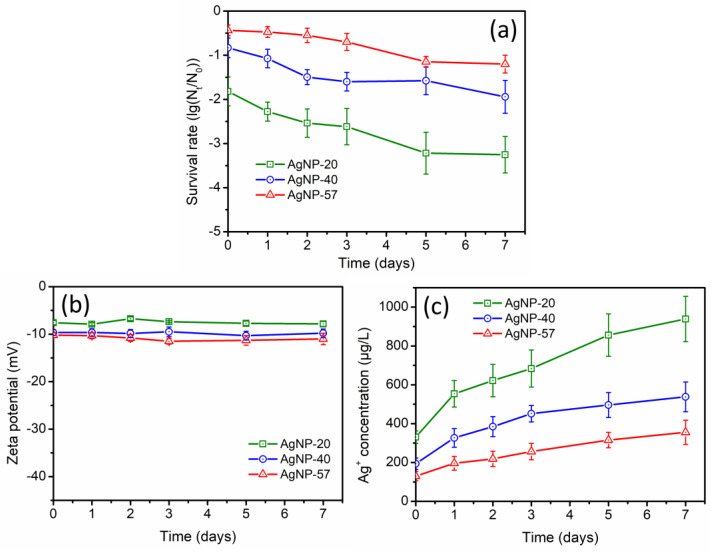
(**a**) Changes in antibacterial activity of different−sized AgNPs during the incubation with ultrapure water (AgNPs samples were diluted to 0.8 mg/L); (**b**) changes in zeta potential of AgNPs and (**c**) Ag^+^ concentrations of suspensions during the incubation with ultrapure water.

During long-term incubation in ultrapure water, the changes in antibacterial activity of different-sized AgNPs were studied. As displayed in Figure 2a, the antibacterial activity of AgNPs was obviously enhanced, with a greater enhancement observed as the AgNPs size decreased. The direct interaction between AgNPs and bacteria, as well as the release of Ag^+^ by AgNPs, were considered to be the two basic pathways by which AgNPs exhibited antibacterial effects [37]. Hence, during the 7-day incubation, the zeta potential values of AgNPs and the Ag^+^ concentration in the suspensions were monitored. According to Figure 2b, the zeta potential of AgNPs remained stable, with almost no noticeable changes, suggesting that the direct interaction between AgNPs and bacteria was hardly affected. However, the Ag^+^ concentration in the suspensions gradually increased during the long-term incubation (Figure 2c), which could be considered the main reason for the enhanced antibacterial activity. In ultrapure water, AgNPs constantly dissolved and released Ag^+^ due to the oxidation by dissolved oxygen. After 7 days of incubation, the Ag^+^ concentrations of AgNP-20, AgNP-40 and AgNP-57 increased by 607.7, 344.0 and 225.8 μg/L, respectively. Smaller-sized AgNPs possess a larger specific surface area and higher surface activity, making them more prone to oxidation and to releasing more Ag^+^. This could also account for the more pronounced increase in the antibacterial activity of the smaller-sized AgNPs during the 7-day incubation. Additionally, it was also found that the increase rate of the Ag^+^ concentration gradually slowed down with the extension of the incubation time (Figure 2c). Studies have shown that as the amount of Ag^+^ increased, Ag^+^ gradually accumulated around AgNPs, which suppressed the dissolution of the Ag_2_O layer on their surface, thereby inhibiting the release of Ag^+^ and the further dissolution of AgNPs [38,39].

### 2.3. The Effect of NO_3_^−^

**Figure 3:**
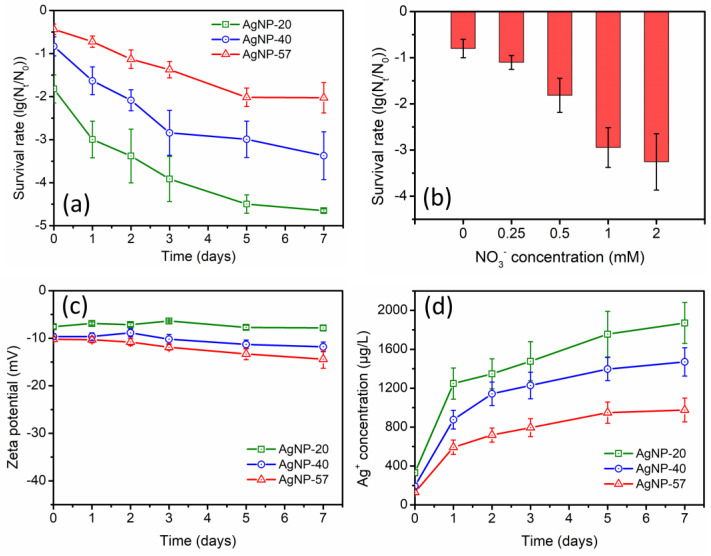
(**a**) Changes in antibacterial activity of different−sized AgNPs during the incubation with 0.5 mM NO_3_^−^ (AgNPs samples were diluted to 0.8 mg/L); (**b**) effect of NO_3_^−^ concentration on the antibacterial activity of AgNP−40 (AgNPs samples were diluted to 0.8 mg/L); (**c**) changes in zeta potential of AgNPs and (**d**) Ag^+^ concentrations of suspensions during the incubation with 0.5 mM NO_3_^−^.

During the incubation with 0.5 mM NO_3_^−^, significantly enhanced antibacterial activity of AgNPs was observed, and the enhancement was more pronounced for the smaller-sized AgNPs (Figure 3a). Moreover, it was found that the enhancement of the antibacterial activity in NO_3_^−^ solution was greater compared to that in ultrapure water (Figure 2a). For example, after 3 days of incubation, the antibacterial activity of AgNP-20 in ultrapure water was 2.6-log, while in NO_3_^−^ solution, it was 3.9-log. In addition, the effect of NO_3_^−^ concentration (0, 0.25, 0.5, 1.0 and 2.0 mM) was also investigated. Based on Figure 3b, the enhancement of the antibacterial activity of AgNP-40 was more significant with increasing NO_3_^−^ concentration. It can be seen that the introduction of NO_3_^−^ greatly enhanced the antibacterial activity of AgNPs during the incubation.

The zeta potential and Ag^+^ concentration of AgNPs suspensions were also determined during the 7-day incubation in 0.5 mM NO_3_^−^ solution. No remarkable changes were observed in the zeta potential (Figure 3c), whereas the Ag^+^ concentration increased substantially (Figure 3d). After incubation for 7 days, the Ag^+^ concentrations of AgNP-20, AgNP-40 and AgNP-57 increased by 1539.6, 1276.7 and 846.4 μg/L, respectively. As the particle size decreased, the increase in Ag^+^ concentration became more pronounced. Furthermore, compared to ultrapure water, the increase in Ag^+^ concentration was greater in NO_3_^−^ solution, which could also be the reason for the more pronounced enhancement of the antibacterial activity. Studies have indicated that the introduction of electrolytes could facilitate AgNPs dissolution [28,40]. In ultrapure water (without electrolytes), Ag^+^ would accumulate around AgNPs and inhibit the dissolution of the Ag_2_O layer on their surfaces [38,39]. However, with the introduction of electrolytes, the accumulated Ag^+^ would redistribute, or it would be displaced by electrolyte ions (such as Na^+^ and K^+^), thereby promoting the dissolution of the Ag_2_O layer. Simultaneously, this process also exposed the easily oxidizable metallic silver nuclei to the external environment, promoting the further oxidation and dissolution of AgNPs [21,39]. Hence, the significant increase in the Ag^+^ concentration and the resulting enhancement of the antibacterial activity should be attributed to the presence of electrolytes. However, throughout the incubation process, NO_3_^−^ itself seemed to have no direct interaction with AgNPs and did not exert any specific effects on AgNPs. 

### 2.4. The Effect of SO_4_^2−^

**Figure 4:**
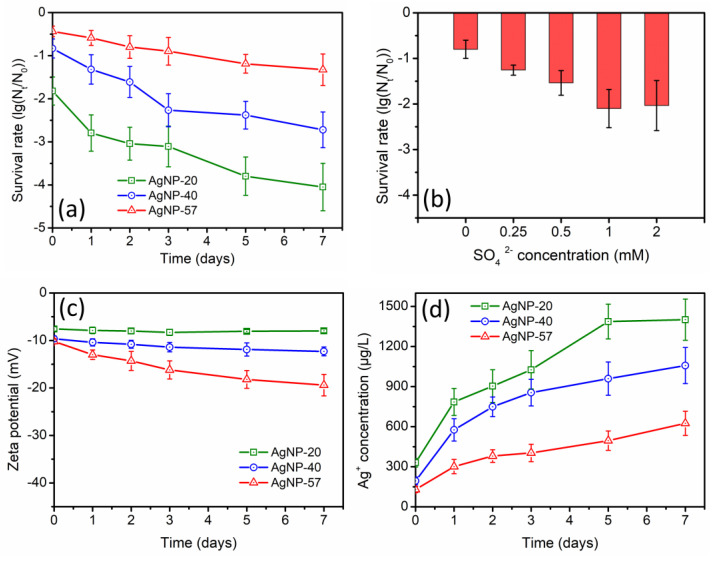
(**a**) Changes in antibacterial activity of different−sized AgNPs during the incubation with 0.5 mM SO_4_^2−^ (AgNPs samples were diluted to 0.8 mg/L); (**b**) effect of SO_4_^2−^ concentration on antibacterial activity of AgNP−40 (AgNPs samples were diluted to 0.8 mg/L); (**c**) changes in zeta potential of AgNPs and (**d**) Ag^+^ concentrations of suspensions during the incubation with 0.5 mM SO_4_^2−^.

The variations in the antibacterial activity of AgNPs during the incubation with 0.5 mM SO_4_^2−^ were exhibited in Figure 4a. A remarkable enhancement of the antibacterial activity was also observed, and the enhancement was greater for the smaller-sized AgNPs. Furthermore, it could be observed that the enhancement of the antibacterial activity in SO_4_^2−^ solution was greater than that in ultrapure water (Figure 2a), but smaller than that in NO_3_^−^ solution (Figure 3a). Additionally, the effect of SO_4_^2−^ concentration was depicted in Figure 4b. Within the concentration range of 1.0 mM, increasing SO_4_^2−^ concentration led to a more obvious enhancement of the antibacterial activity of AgNP-40. Nevertheless, with SO_4_^2−^ concentration increased to 2.0 mM, the antibacterial activity of AgNP-40 was not further enhanced; instead, it slightly decreased compared to that at 1.0 mM concentration. It seemed that high concentrations of SO_4_^2−^ exerted an inhibitory effect on the antibacterial activity of AgNPs.

Figure 4c displayed the variations in the zeta potential values of AgNPs. During the 7-day incubation with 0.5 mM SO_4_^2−^, the zeta potential of AgNP-20 did not show obvious changes, while the zeta potential values of AgNP-40 and AgNP-57 exhibited a slight decrease (with more negative charges), respectively, decreasing from −9.6 mV and −10.2 mV to −12.3 mV and −19.4 mV. The presence of SO_4_^2−^ was probably responsible for this decrease in zeta potential. Meanwhile, it was worth noting that the variation in the zeta potential of AgNPs was particle-size dependent. With increasing particle size, the decrease in zeta potential was more apparent. It seemed that the large-sized AgNPs were more likely to interact with SO_4_^2−^, thereby altering the surface properties of AgNPs.

During the 7-day incubation with 0.5 mM SO_4_^2−^, the Ag^+^ concentrations of AgNP-20, AgNP-40 and AgNP-57 increased by 1069.8, 863.5 and 495.3 μg/L, respectively (Figure 4d). It was observed that the increase of Ag^+^ concentration in SO_4_^2−^ solution was larger than that in ultrapure water (Figure 2c), but smaller than that in NO_3_^−^ solution (Figure 3d), which was consistent with the results of the antibacterial experiments. Under the same concentration of anion, the ionic strength of SO_4_^2−^ solution was higher than that of NO_3_^−^ solution. Thus, a more significant dissolution of AgNPs would be expected, and the Ag^+^ concentration in SO_4_^2−^ solution was supposed to be higher than that in NO_3_^−^ solution. However, the actual results were exactly the opposite. Hence, it was speculated that SO_4_^2−^ might exert specific effects on AgNPs, resulting in a certain inhibitory effect on the dissolution of AgNPs.

The antibacterial activity of AgNPs was correlated with their surface potential. Amro et al. conducted a comparative study on the toxicity of AgNPs with different surface potentials, and the results indicated that an increase in negative charge on the particle surface resulted in a decrease in the toxicity of AgNPs [41]. After incubation with SO_4_^2−^, the zeta potential of AgNPs decreased, causing an enhancement of the electrostatic repulsion between bacteria and AgNPs, which, in turn, somewhat weakened the antibacterial activity of AgNPs. Nevertheless, the introduction of SO_4_^2−^ also promoted the dissolution of AgNPs, and the Ag^+^ concentration in the suspensions noticeably increased. Therefore, the antibacterial activity of AgNPs was still notably elevated during the incubation with SO_4_^2−^.

### 2.5. The Effect of HCO_3_^−^/CO_3_^2−^

**Figure 5:**
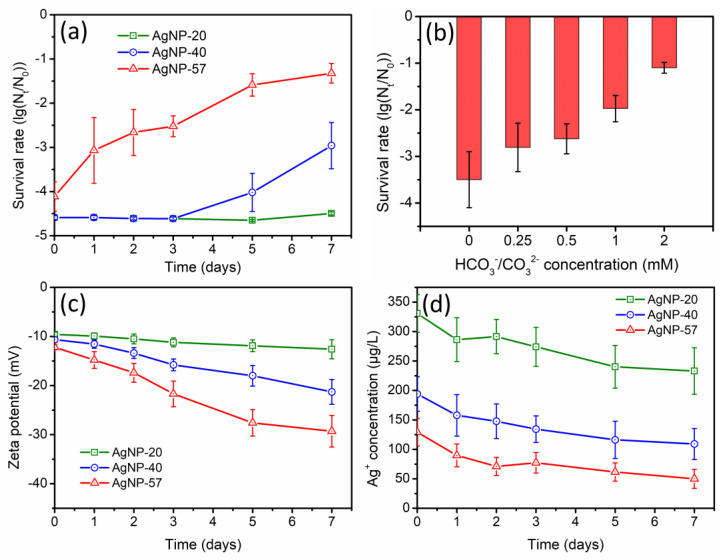
(**a**) Changes in antibacterial activity of different−sized AgNPs during the incubation with 0.5 mM HCO_3_^−^/CO_3_^2−^ (AgNPs samples were diluted to 4.8 mg/L); (**b**) effect of HCO_3_^−^/CO_3_^2−^ concentration on antibacterial activity of AgNP−40 (AgNPs samples were diluted to 3.2 mg/L); (**c**) changes in zeta potential of AgNPs and (**d**) Ag^+^ concentrations of suspensions during the incubation with 0.5 mM HCO_3_^−^/CO_3_^2−^.

Based on Figure 5a, the antibacterial activity of AgNPs was notably weakened, in stark contrast to the results obtained in NO_3_^−^ and SO_4_^2−^ solutions (Figure 3a and Figure 4a). After 7 days of incubation with 0.5 mM HCO_3_^−^/CO_3_^2−^, the antibacterial activity of AgNP-20, AgNP-40 and AgNP-57 decreased by 0.1-log, 1.6-log and 2.8-log, respectively. It was observed that the decline in the antibacterial activity of AgNPs was more pronounced as the particle size increased. The larger-sized AgNPs seemed to be more significantly affected by HCO_3_^−^/CO_3_^2−^. In addition, the influence of HCO_3_^−^/CO_3_^2−^ concentration was depicted in Figure 5b. As the concentration of HCO_3_^−^/CO_3_^2−^ increased, the inhibitory effect on the antibacterial activity of AgNP-40 became more remarkable.

The variation in the zeta potential was displayed in Figure 5c. During the 7-day incubation with 0.5 mM HCO_3_^−^/CO_3_^2−^, the zeta potential values of different-sized AgNPs all decreased. The introduction of HCO_3_^−^/CO_3_^2−^ increased the pH value of the solution. Bojie et al. investigated the impact of pH in HCO_3_^−^/CO_3_^2−^ solutions on the zeta potential of AgNPs, and the results indicated that the decrease in zeta potential was caused by the presence of HCO_3_^−^/CO_3_^2−^ rather than the increase of pH [29]. After 7 days of incubation, the zeta potential of AgNP-20, AgNP-40 and AgNP-57 decreased by 3.0, 10.6 and 17.1 mV, respectively. It was observed that the decrease in the zeta potential was more significant with the increase of particle size, similar to, but more pronounced than, the changes observed in SO_4_^2−^ solution (Figure 4c). 

Figure 5d exhibited the variation of the Ag^+^ concentration. Contrary to the phenomena observed in NO_3_^−^ and SO_4_^2−^ solutions, the introduction of HCO_3_^−^/CO_3_^2−^ resulted in an obvious decrease in Ag^+^ concentration during the 7-day incubation. This suggested that HCO_3_^−^/CO_3_^2−^ clearly suppressed AgNPs dissolution and consumed the dissolved silver initially presented in the suspensions. After 7 days of incubation, the Ag^+^ concentrations of AgNP-20, AgNP-40 and AgNP-57 decreased by 97.8, 84.7 and 79.1 μg/L, respectively, with relatively minor differences. However, the initial Ag^+^ concentrations in AgNP-20, AgNP-40 and AgNP-57 suspensions were 330.9, 193.9 and 129.1 μg/L, respectively. Hence, the Ag^+^ concentrations of AgNP-20, AgNP-40 and AgNP-57 decreased by 29.5%, 43.7% and 61.3%, respectively, after 7 days of incubation. It can be observed that the decrease ratio of the Ag^+^ concentration increased as the particle size increased, indicating a greater impact of HCO_3_^−^/CO_3_^2−^ on the large-sized AgNPs.

During the long-term incubation with HCO_3_^−^/CO_3_^2−^, the larger-sized AgNPs exhibited a greater decrease in zeta potential, enhancing the electrostatic repulsion between AgNPs and bacteria. Furthermore, the effects of HCO_3_^−^/CO_3_^2−^ on dissolved silver were more pronounced in the case of larger-sized AgNPs, causing a higher decrease ratio in the Ag^+^ concentration. As a result, the inhibitory effect of HCO_3_^−^/CO_3_^2−^ on the antibacterial activity of AgNPs became more evident as the particle size increased.

### 2.6. The Effect of Cl^−^

**Figure 6:**
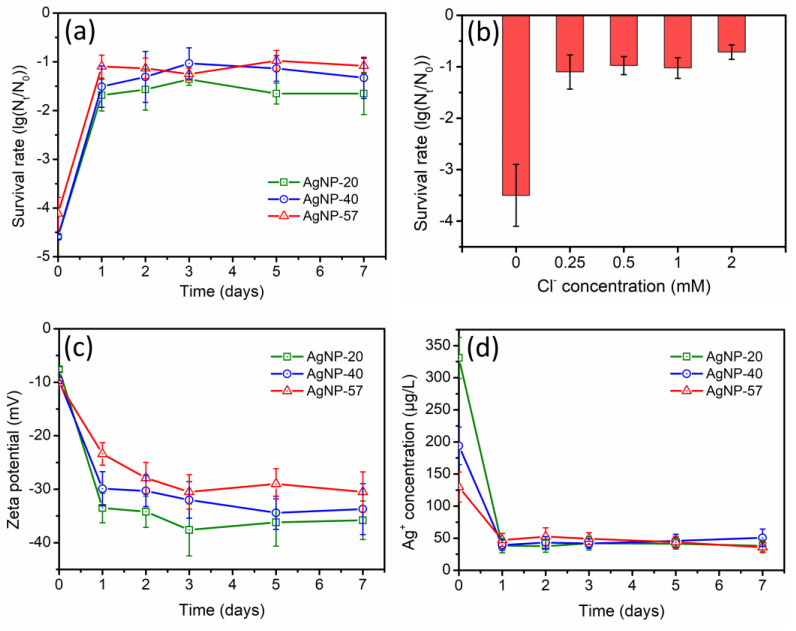
(**a**) Changes in antibacterial activity of different−sized AgNPs during the incubation with 0.5 mM Cl^−^ (AgNPs samples were diluted to 4.8 mg/L); (**b**) effect of Cl^−^ concentration on antibacterial activity of AgNP−40 (AgNPs samples were diluted to 3.2 mg/L); (**c**) changes in zeta potential of AgNPs and (**d**) Ag^+^ concentrations of suspensions during the incubation with 0.5 mM Cl^−^.

According to Figure 6a, the antibacterial activity of AgNPs was greatly inhibited after exposure to Cl^−^ (0.5 mM), and this inhibitory effect was more pronounced than that observed in HCO_3_^−^/CO_3_^2− (^Figure 5a). In addition, the particle size did not exhibit an obvious impact on the variation of the antibacterial activity, as different-sized AgNPs displayed similar declining trends. And the decline in the antibacterial activity nearly stabilized after only 1 day of incubation. Figure 6b demonstrated the influence of Cl^−^ concentration, showing that the inhibitory effect on the antibacterial activity of AgNP-40 was not further increased with higher Cl^−^ concentrations.

During the 7-day incubation with 0.5 mM Cl^−^, the variation in the zeta potential was shown in Figure 6c. The zeta potential values of different-sized AgNPs all experienced significant decreases. Thus, the interaction between AgNPs and bacteria was greatly hindered. Furthermore, the Ag^+^ concentration in the suspensions of different-sized AgNPs also exhibited a drastic decline (Figure 6d), reaching a relatively stable level after only 1 day of incubation. Consequently, the antibacterial activity of AgNPs sharply decreased (Figure 6a).

### 2.7. Discussion

The above experimental results indicated that anions in water significantly affected the antibacterial activity and physicochemical properties of AgNPs. The introduction of anions led to the addition of electrolytes, which promoted the dissolution of AgNPs. This was the common effect that the introduction of anions would have on AgNPs. On the other hand, the anions also exerted specific effects on AgNPs, altering their surface properties and dissolution characteristics. Our results suggested that the specific effects of anions on AgNPs gradually increased in the order of NO_3_^−^ < SO_4_^2−^ < HCO_3_^−^/CO_3_^2−^ < Cl^−^, as evidenced by the more pronounced decline in zeta potential and the more significant inhibition in AgNPs dissolution. Levard et al. found that AgCl layer formed on AgNPs surface after treatment with Cl^−^ solution [27], which resulted in an obvious decrease in the zeta potential of AgNPs [27,35,42]. Additionally, this passivating layer covered on AgNPs surface was also reported to significantly inhibit AgNPs dissolution [43]. Therefore, the specific effects of the anions on AgNPs observed in this study should also be associated with the generation of coating layers on AgNPs surfaces.

NO_3_^−^ does not form insoluble compounds with Ag^+^. No coating layers were formed on AgNPs surfaces. Therefore, no specific effects of NO_3_^−^ on AgNPs were observed, and the zeta potential of AgNPs remained relatively stable during the incubation with NO_3_^−^ (Figure 3c). However, the introduction of electrolytes greatly facilitated the dissolution of AgNPs, causing a significant increase in the Ag^+^ concentration (Figure 3d). Consequently, the antibacterial activity of AgNPs also remarkably increased (Figure 3a).

SO_4_^2−^ can form insoluble compounds with Ag^+^, but with a relatively high solubility product (Ag_2_SO_4_ (*K_sp_* = 1.20 × 10^−5^)). During the incubation with SO_4_^2−^, a small amount of Ag_2_SO_4_ coating layer might be generated on AgNPs surfaces, causing a slight decline in the zeta potential of the particles (Figure 4c) and a mild inhibitory effect on AgNPs dissolution. Nevertheless, the promoting effect of the electrolytes on AgNPs dissolution still predominated, ultimately inducing a noticeable increase in the Ag^+^ concentration (Figure 4d). This, in turn, led to a great enhancement in the antibacterial activity of AgNPs (Figure 4a).

For HCO_3_^−^/CO_3_^2−^, the solubility of its corresponding silver-based product, Ag_2_CO_3_ (*K_sp_* = 8.46 × 10^−12^), was significantly lower than that of Ag_2_SO_4_ (*K_sp_* = 1.20 × 10^−5^). Therefore, much more coating layer (Ag_2_CO_3_ layer) might be formed on AgNPs surfaces, causing a more obvious decline in zeta potential of the particles (Figure 5c) and a more notable inhibition in AgNPs dissolution. HCO_3_^−^/CO_3_^2−^ exhibited a more substantial specific effect on AgNPs than SO_4_^2−^. As a considerable amount of Ag_2_CO_3_ layer was coated on AgNPs surfaces, the facilitating effect of the electrolytes on AgNPs dissolution was no longer dominant. Moreover, the formation of Ag_2_CO_3_ layer might consume the dissolved silver that initially presented in this system. As a result, the Ag^+^ concentration in the suspensions obviously decreased during the incubation with HCO_3_^−^/CO_3_^2−^ (Figure 5d). Ultimately, the decreasing zeta potential and Ag^+^ concentration caused a reduction in the antibacterial activity of AgNPs (Figure 5a). 

Cl^−^ can readily form insoluble substances with Ag^+^ (AgCl (*K_sp_* = 1.77 × 10^−10^)), and the solubility of AgCl is lower than that of Ag_2_CO_3_. Therefore, the specific effect of Cl^−^ on AgNPs was found to be more pronounced than that of HCO_3_^−^/CO_3_^2−^. Within a relatively short incubation time, a large quantity of AgCl coating layer might be formed on AgNPs surfaces, resulting in a drastic decline in the zeta potential (Figure 6c), Ag^+^ concentration (Figure 6d) and antibacterial activity of AgNPs (Figure 6a). 

Overall, it could be concluded that the lower the solubility of the silver-based product corresponding to the anion, the easier it was to form coating layers on AgNPs surfaces during the incubation with the anion, thereby causing a decline in the zeta potential of the particles (with more negative charges), inhibition of AgNPs dissolution and reduction in the antibacterial activity of AgNPs.

During the incubation process, the variations in the physicochemical properties of AgNPs were also found to correlate with the particle size. In NO_3_^−^ and SO_4_^2−^ solutions, the promoting effect of the electrolytes on AgNPs dissolution was predominant. Therefore, the smaller-sized nanoparticles with larger specific surface areas presented a significant advantage, resulting in a more pronounced increase in the Ag^+^ concentration (Figure 3d and Figure 4d). However, in HCO_3_^−^/CO_3_^2−^ solution, AgNPs dissolution was greatly inhibited, and the Ag^+^ concentration was markedly reduced, with a higher decrease ratio in Ag^+^ concentration for the larger-sized AgNPs (Figure 5d). In terms of the zeta potential, as AgNPs size increased, a more pronounced decrease in the zeta potential was observed in SO_4_^2−^ and HCO_3_^−^/CO_3_^2−^ solutions (Figure 4c and Figure 5c). This implied that the anions exerted stronger specific effects on the larger-sized AgNPs, leading to the generation of more coating layers on their surfaces. During the incubation with Cl^−^, the particle size did not exhibit an obvious impact on the variations in the physicochemical properties of AgNPs. The zeta potential and Ag^+^ concentrations of different-sized AgNPs displayed a similarly sharp decrease (Figure 6c,d), which might be attributed to the rapid generation of AgCl coatings on AgNPs surfaces caused by the strong interaction of Cl^−^ with Ag^+^.

**Figure 7:**
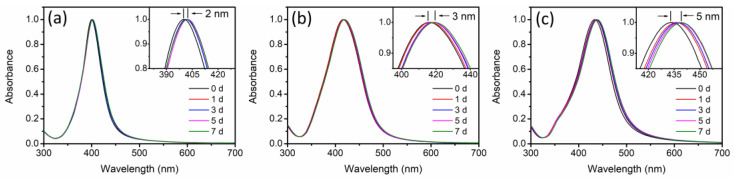
Changes in UV-visible spectrum of AgNPs during the incubation with 0.5 mM HCO_3_^−^/CO_3_^2−^. ((**a**) AgNP−20, (**b**) AgNP−40, (**c**) AgNP−57).

**Figure 8:**
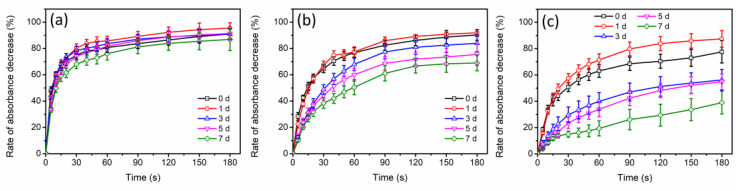
The decrease rate of AgNPs (incubated with 0.5 mM HCO_3_^−^/CO_3_^2−^) absorbance over time after the introduction of H_2_O_2_. ((**a**) AgNP−20, (**b**) AgNP−40, (**c**) AgNP−57).

**Figure 9:**
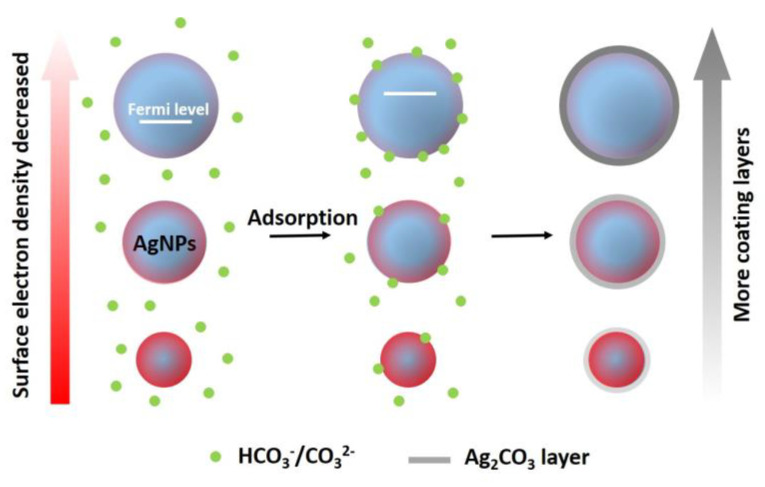
Speculated schematic diagram showing the interaction of different−sized Ag NPs with HCO_3_^−^/CO_3_^2−^.

To further investigate the specific effects of the anions on different-sized AgNPs, the generation of surface coating layers on the particles was performed during the long-term incubation. The aforementioned experimental results indicated that SO_4_^2−^ had a weak specific effect on AgNPs, while the specific effect of Cl^−^ was too strong. Therefore, HCO_3_^−^/CO_3_^2−^ was chosen for this experimental study. The formation of an Ag_2_CO_3_ layer has been found to alter the optical properties of AgNPs, causing a red shift in the absorbance peak of the UV-visible spectra of AgNPs [29]. Hence, during the 7-day incubation with HCO_3_^−^/CO_3_^2−^, the UV-visible spectra of different-sized AgNPs were collected. Based on Figure 7, the absorbance peaks of different-sized AgNPs showed continuous red shifts. After 7 days of incubation, the absorbance peaks of AgNP-20, AgNP-40 and AgNP-57 were red-shifted by 2, 3 and 5 nm, respectively. A greater red shift in the absorbance peak was observed in the larger-sized AgNPs, indicating that a larger amount of Ag_2_CO_3_ coating layer was generated. In addition, the Ag_2_CO_3_ layer, acting as a passivating layer, has also been demonstrated to prevent AgNPs from oxidation and dissolution [29]. Hence, to further verify the generation of Ag_2_CO_3_ layer on different-sized AgNPs, their dissolution behaviors under H_2_O_2_ oxidation were investigated. After the introduction of H_2_O_2_, the changes in absorbance at the maximum absorption wavelengths of different-sized AgNPs was recorded, in order to reflect the dissolution behavior of AgNPs in a qualitative way. As displayed in Figure 8a, AgNP-20 only exhibited a slight resistance to the oxidation by H_2_O_2_ after incubation with HCO_3_^−^/CO_3_^2−^ for 7 days. For AgNP-40, obvious antioxidant ability was gradually exhibited with the incubation time prolonged, as evidenced by the notable suppression of absorbance decay. After 7 days of incubation, the rate of absorbance decay decreased from 90.3% to 69.0% after reacting with the H_2_O_2_ for 180 s (Figure 8b). Compared to AgNP-40, stronger resistance to oxidation was observed in AgNP-57, with the rate of absorbance decay decreasing from 77.6% to 39.0% (Figure 8c). Hence, the larger-sized AgNPs displayed stronger antioxidant ability after long-term incubation with HCO_3_^−^/CO_3_^2−^, indicating that more Ag_2_CO_3_ layers were formed on the particle surfaces. This corresponded with the results of the UV-visible spectrum (Figure 7) and zeta potential (Figure 5c). Additionally, according to Figure 8c, the oxidation of AgNP-57 by H_2_O_2_ was found to be facilitated after 1 day of incubation. It has been demonstrated that the adsorption of nucleophiles on AgNPs would induce an upward shift in the Fermi level, making it easier for AgNPs to lose electrons and be oxidized [39]. HCO_3_^−^/CO_3_^2−^, as a nucleophilic reagent, can adsorb onto the surface Ag atoms of AgNPs by sharing a pair of electrons with the unoccupied orbital of Ag. Hence, the facilitation in the oxidation of AgNP-57 by H_2_O_2_ should be attributed to the adsorption of HCO_3_^−^/CO_3_^2−^. However, in AgNP-20 and AgNP-40, no obvious promotion in the oxidation was observed (Figure 8a,b), implying that the adsorption of HCO_3_^−^/CO_3_^2−^ on the particle surface was not notable. It seemed that the adsorption of HCO_3_^−^/CO_3_^2−^ was also size-dependent, and that it was more prone to adsorbing onto larger-sized AgNPs. As characterized by the UV-visible spectroscopy (Figure 7), the larger-sized AgNPs absorbed light with longer wavelengths and lower energy, suggesting a lower electron density on the surfaces of the particles [34]. Therefore, we speculated that the adsorption of HCO_3_^−^/CO_3_^2−^ might be influenced by the surface electron density of particles. As depicted in Figure 9, larger-sized AgNPs with lower surface electron density might be more conducive to complexing with HCO_3_^−^/CO_3_^2−^, thus promoting the adsorption of HCO_3_^−^/CO_3_^2−^. The adsorption of HCO_3_^−^/CO_3_^2−^ led to the accumulation of HCO_3_^−^/CO_3_^2−^ on the surfaces of the larger-sized AgNPs, thereby facilitating the generation of coating layers and altering the physicochemical properties of the particles.

## 3. Materials and Methods

### 3.1. Materials

Sodium sulfate, sodium nitrate, sodium bicarbonate, sodium chloride and hydrogen peroxide (H_2_O_2_) were provided by Aladdin Chemistry Co., Ltd. (Shanghai, China). Nutrient agar and nutrient broth were provided by BD Biosciences (Franklin Lakes, NJ, USA). Every reagent was analytically pure and utilized with no additional purification. Ultrapure water (Aquaplore 2S, Wilmington, DE, USA) was utilized throughout the experiment. 

### 3.2. Bacteria

*Escherichia coli* (*E. coli*, ATCC 25922), a gram-negative bacterial strain, was selected as the model microorganism for antibacterial experiments. It was cultured in 100 mL nutrient broth and incubated overnight at 37 °C in a constant temperature shaker incubator. The sample was then centrifuged at a centrifugal force of 10,000× *g* for 2 min, washed thrice with ultrapure water to eliminate excessive impurities, and finally resuspended to make the bacteria stock suspension.

### 3.3. Synthesis and Characterization of AgNPs

Through the approach depicted by Bastus et al. [44], the synthesis of PVP-coated AgNPs with varying sizes were performed. Next, the morphology of synthesized AgNPs was characterized using transmission electron microscopy (TEM, JEM-2011, Jeol, Tokyo, Japan). Inductively coupled plasma mass spectrometry (ICP-MS, Agilent-7700, Santa Clara, CA, USA) was utilized to measure the concentrations of synthesized AgNPs. Prior to ICP-MS analysis, the AgNPs samples underwent digestion with concentrated HNO_3_ for a duration of 2 h. The zeta potentials of AgNPs were measured by a Zetasizer Nano instrument (Malvern Instruments, Malvern, UK).

### 3.4. Experimental Procedure

The stock suspensions of different-sized AgNPs (AgNP-20, AgNP-40, AgNP-57) were diluted to 12.8 mg/L using 0.5 mM solutions of NO_3_^−^, SO_4_^2−^, HCO_3_^−^/CO_3_^2−^ and Cl^−^, respectively. Additionally, ultrapure water was used as a control without the influence of additional anions. Next, the AgNPs dilutions were incubated under dark condition at 25 °C. At specified time intervals (days 0, 1, 2, 3, 5 and 7), samples were taken to measure the zeta potential, dissolved silver concentration and antibacterial activity. Additionally, in this study, the impact of anion concentration on AgNPs was also investigated. The stock suspension of AgNP-40 was diluted to 12.8 mg/L with different concentrations (0, 0.25, 0.5, 1.0 and 2.0 mM) of NO_3_^−^, SO_4_^2−^, HCO_3_^−^/CO_3_^2−^ and Cl^−^ solutions, respectively. After a 2-day incubation at 25 °C in a dark environment, samples were taken and diluted to appropriate concentration for antibacterial experiments. Each experiment was conducted three times.

Dissolved silver in suspensions was separated by Amicon Ultra-15 3K ultrafiltration centrifuge tubes (Millipore, Billerica, MA, USA). The chosen samples were placed into the ultrafiltration centrifuge tubes and then subjected to centrifugation at 15,000 rpm for 10 min. The nominal pore size of the porous cellulose membrane in the ultrafiltration centrifuge tube was between 1 and 2 nm. This size was sufficiently small to filter out all AgNPs from the sample. After digestion with HNO_3_, the content of Ag^+^ in obtained solutions was measured using ICP-MS.

Bacterial suspensions were mixed with AgNPs samples for antibacterial experiments, with the initial concentration of bacteria set at 10^6^ CFU/mL. Following 30 min of treatment with AgNPs, samples were taken for serial 10-fold dilutions. Next, 30 μL of each dilution was plated onto nutrient agar plates and spread evenly. After overnight incubation at 37 °C in a constant-temperature incubator, the colony-forming units (CFUs) on the plates were counted. The calculation of the bacterial survival rate was set to be lg(N_t_/N_0_), in which N_t_ and N_0_ represented the remaining and initial concentrations of viable bacteria (CFU/mL), respectively. All tests were conducted in triplicate.

A UV-3600 spectrophotometer (Thermo Fisher Scientific, Waltham, MA, USA) was used to collect the UV-visible spectra (with a wavelength range from 300 to 700 nm) of AgNPs suspensions (with the total Ag concentration controlled at 6.4 mg/L). The wavelength of maximum absorption was also obtained. Subsequently, 200 g/L H_2_O_2_ was added into the AgNPs suspensions for antioxidant experiments, and the changes in absorbance were monitored at the wavelength of maximum absorption.

## 4. Conclusions

In aquatic environments, the fate and behavior of AgNPs are inevitably affected by anions. In this study, we investigated the alterations in the antibacterial activity and physicochemical properties of different-sized AgNPs (20, 40 and 57 nm) in response to the influence of anions (NO_3_^−^, SO_4_^2−^ and HCO_3_^−^/CO_3_^2−^, Cl^−^). Additionally, the impact of anion characteristics and particle size on the interactions between AgNPs and anions were also revealed. The results suggested that the lower the solubility of the silver-based product corresponding to the anion, the more likely it was to generate a passivating layer on AgNPs surfaces during the incubation with the anions, which, in turn, inhibited the dissolution of AgNPs and decreased the zeta potential of particles (which became more negative). As a result, the antibacterial activity of AgNPs was reduced. In addition, the particle size was also found to be a crucial factor influencing the interaction of AgNPs with anions. During the incubation with the anions, the inhibition of the dissolution and the decrease in the zeta potential were more pronounced in the larger-sized AgNPs. Through H_2_O_2_-mediated oxidation experiments, it was found that the anions were more prone to adsorbing onto the larger-sized AgNPs. With the adsorption of anions, the formation of passivating layers on AgNPs surfaces was facilitated, subsequently inducing more pronounced variations in the physicochemical properties and antibacterial activity of the larger-sized AgNPs. This suggested that the larger-sized AgNPs were more prone to experiencing specific effects from anions.

## Data Availability

The original contributions presented in this study are included in the article; further inquiries can be directed to the corresponding author.
